# Unusual Presentation of Reversible Transient Vision Loss After Caesarean Section Under Subarachnoid Block: A Case Report

**DOI:** 10.7759/cureus.52522

**Published:** 2024-01-18

**Authors:** Abhishek Chatterjee, Pratap Rudra Mahanty, Deb Sanjay Nag, Nilanjan Sarkar

**Affiliations:** 1 Anaesthesiology, Tata Main Hospital, Jamshedpur, IND; 2 Radiology, Tata Main Hospital, Jamshedpur, IND

**Keywords:** posterior reversible encephalopathy syndrome, caesarean section, pregnancy, pres, loss of vision

## Abstract

A transient vision loss is not commonly encountered during the postoperative period following a caesarean section. Although numerous causes have been suggested for transient vision loss, when loss of vision is associated with seizures and headaches, the differential diagnoses include hemolysis, elevated liver enzymes, low platelet syndrome, reversible cerebral vasoconstriction syndrome, posterior reversible encephalopathy syndrome (PRES), dural venous thrombosis, and central retinal arteriolar occlusion. We report a case of a 35-year-old patient who underwent an elective caesarean section under spinal anaesthesia and developed a headache followed by loss of vision and seizures during the postoperative period. An MRI scan of the brain on the same day revealed subtle hyperintensity in bilateral parieto-occipital lobes in the cortical and subcortical areas and bilateral cerebral hemispheres, which indicates PRES. Rapid and complete resolution of symptoms was observed with supportive treatment. Therefore, prompt suspicion and effective management of PRES are of paramount importance to prevent short- and long-term neurological deficits.

## Introduction

Postoperative vision loss after non-ocular surgery is a rare but devastating complication, with a reported incidence ranging from 0.01 to 1% [1]. Numerous aetiological factors have been described in the literature. Transient vision loss after non-ocular surgeries has been reported after general anaesthesia as well as subarachnoid block. Marcoccia et al. identified the causes of loss of vision in parturients during the perioperative period, which included pre-eclampsia/hemolysis, elevated liver enzymes, low platelet (HELLP) syndrome (which can lead to hypertensive retinopathy, exudative retinal detachment, and cortical blindness), central serous chorioretinopathy, Purtscher-like retinopathy, central retinal arteriolar occlusion (CRAO), dural venous thrombosis, posterior reversible encephalopathy syndrome (PRES), and reversible cerebral vasoconstriction syndrome (RCVS) [2]. However, when vision loss is associated with headaches and seizures during the perioperative period of a pregnant patient, the differential diagnoses are limited to HELLP syndrome, CRAO, PRES, and RCVS. We report a case of sudden bilateral vision loss with headache and seizure post-caesarean section in a normotensive pregnant patient.

## Case presentation

**Figure 1:**
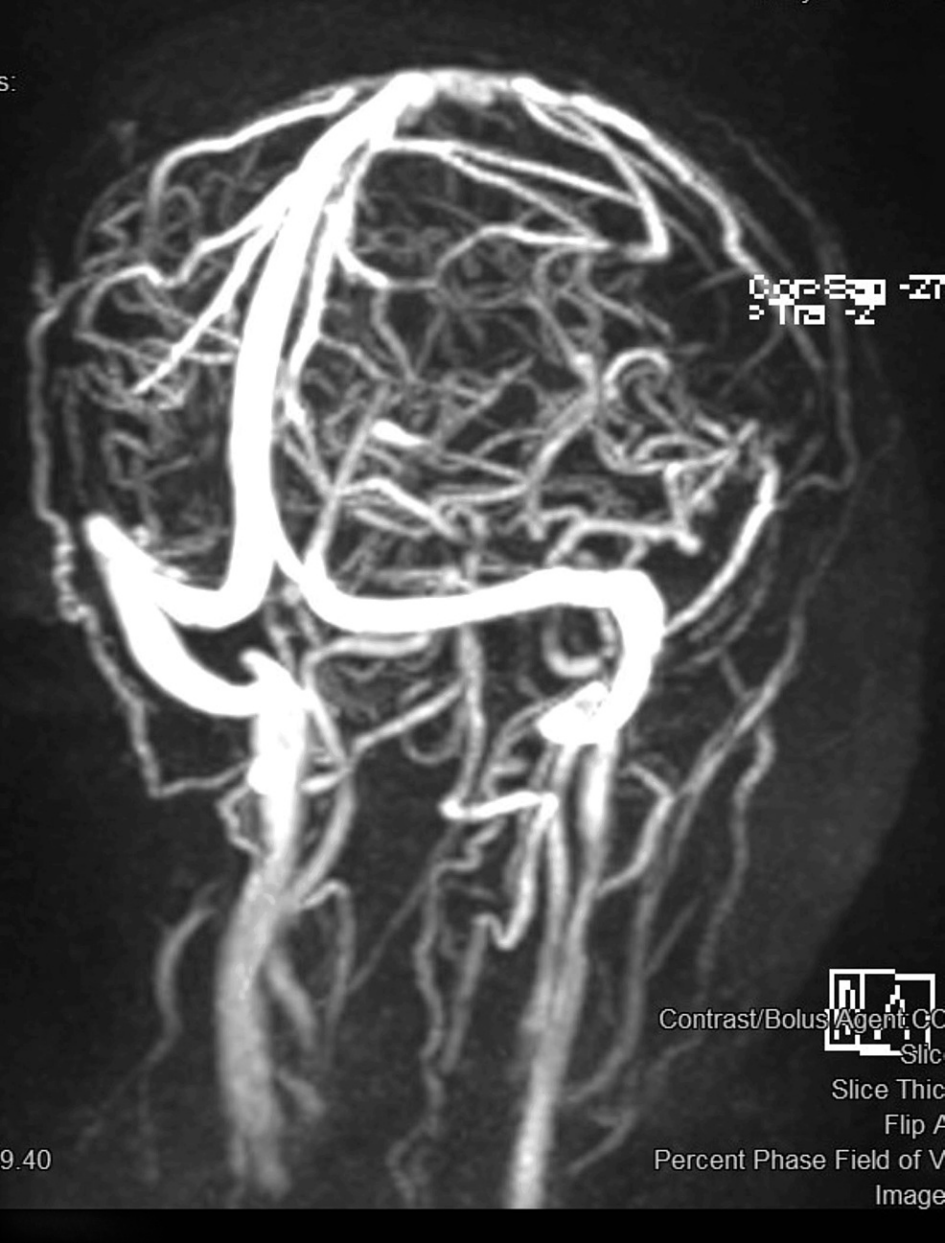
Magnetic resonance (MR) venography: normal study

A 35-year-old gravida 2 patient, at 38 weeks of gestation, was admitted for an elective lower segment caesarean section (LSCS) in view of her bad obstetric history. This patient did not have any co-morbid conditions and therefore underwent an elective LSCS under subarachnoid block. The intraoperative period was uneventful. However, she started complaining of headaches on the second postoperative day, which was initially managed as a post-dural puncture headache. The headache was gradual in onset, mild in intensity, and limited to the parieto-occipital areas. Subsequently, on the fourth postoperative day, she developed sudden loss of vision in both eyes with focal seizures in the periorbital areas and left upper limb. The patient remained hemodynamically stable throughout. Treatment with cefoperazone-sulbactam, levetiracetam, and low-molecular-weight heparin was started, and MR venography, which revealed no abnormalities, was performed (Figure 1).

**Figure 2:**
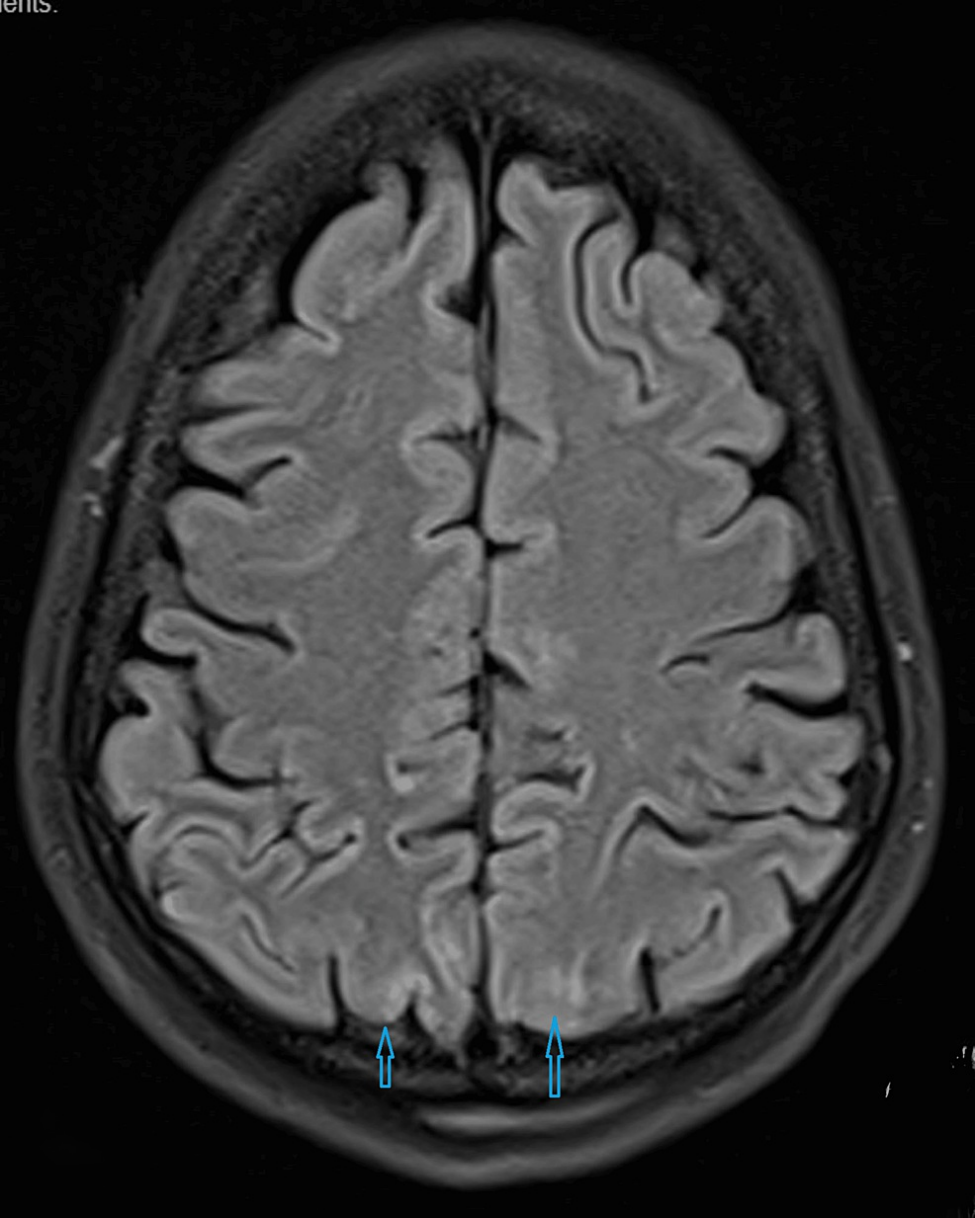
Magnetic resonance imaging (MRI) brain Subtle hyperintensity in bilateral parieto-occipital lobes in cortical and subcortical areas and bilateral cerebral hemispheres indicating posterior reversible encephalopathy syndrome (PRES)

**Figure 3:**
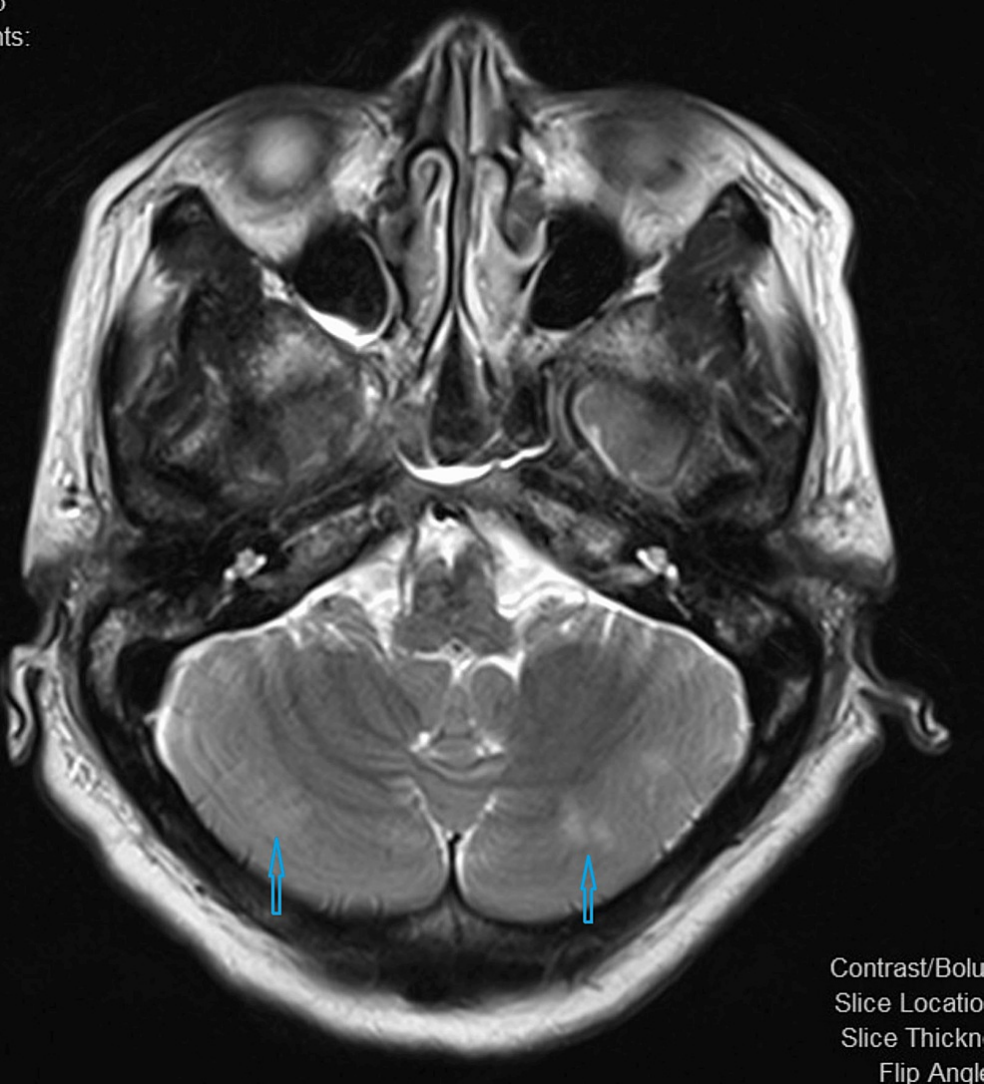
Magnetic resonance imaging (MRI) brain Subtle hyperintensity in bilateral parieto-occipital lobes in cortical and subcortical areas and bilateral cerebral hemispheres indicating posterior reversible encephalopathy syndrome (PRES)

An EEG study also did not show any abnormalities. However, an MRI scan of the brain on the same day showed subtle hyperintensity in the bilateral parieto-occipital lobes in the cortical and subcortical areas and bilateral cerebral hemispheres, which indicates PRES (Figures 2-3).

**Figure 4:**
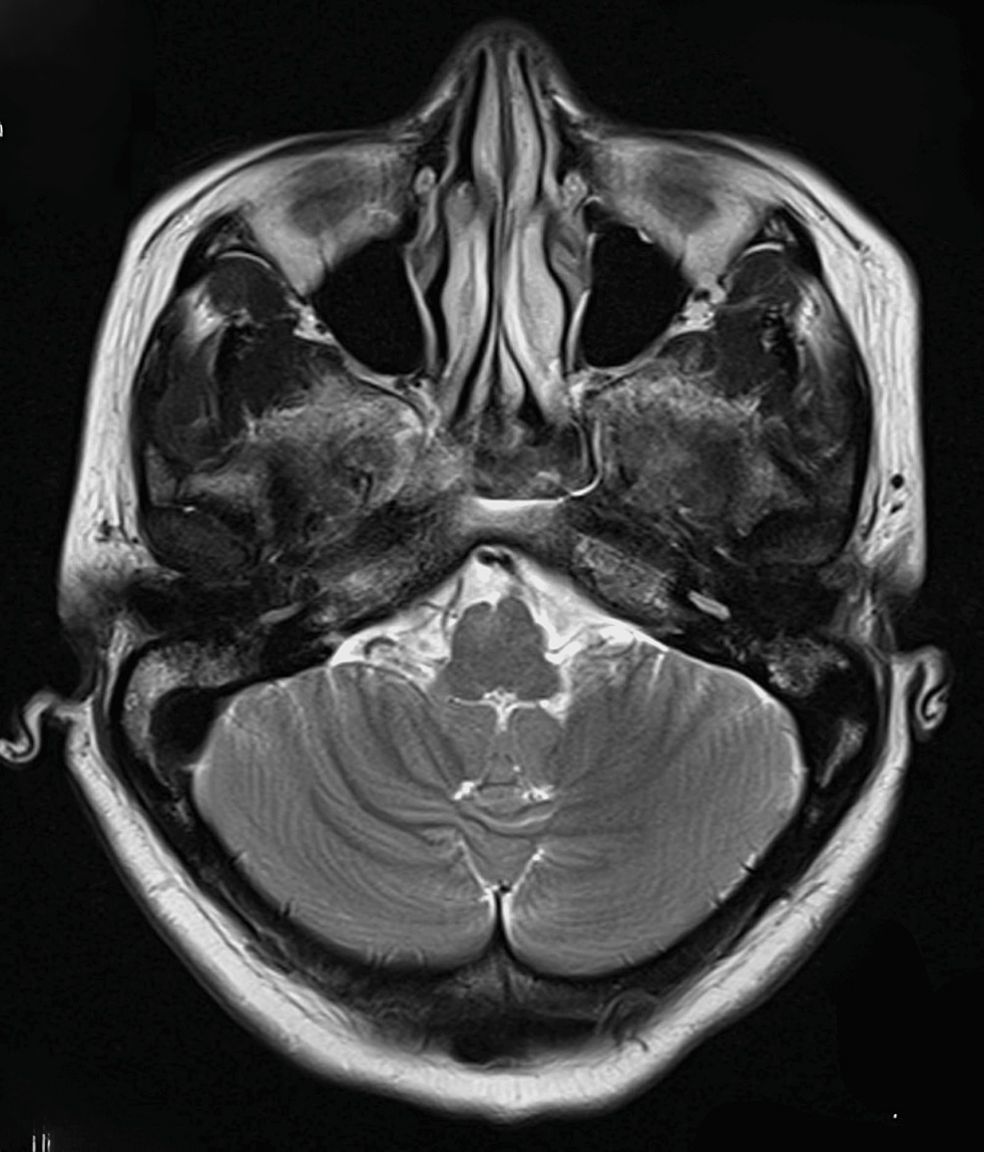
Follow-up magnetic resonance imaging (MRI) brain after two months: normal study

An ophthalmologist also did not find any abnormalities in a detailed eye examination. By the following day, the patient’s vision had improved sufficiently to be able to count fingers, the pupils showed normal reactions to light, and the motor power of all limbs had normalized. The patient’s vision improved on the seventh postoperative day, and she was discharged from the hospital on the tenth postoperative day in a stable condition without any residual vision disturbances. A follow-up MRI scan after two months did not show any abnormalities (Figure 4).

## Discussion

PRES is an entity that can be confirmed clinico-radiologically. Hinchey et al. described it for the first time as a syndrome manifested by headaches, altered mental status, convulsions, and loss of vision [3]. Trikha et al. described PRES as reversible posterior leukoencephalopathy syndrome and also as hyperperfusion encephalopathy [4]. Williams and Wilson, in their study, observed that the autoregulatory mechanism of the cerebral circulation was inhibited when there was an acute rise in blood pressure, which subsequently led to disruption of the blood-brain barrier and ultimately resulted in the development of vasogenic intracerebral edema [5]. The authors noted that the usual location of edema was in the parieto-occipital lobes; however, they also concluded that any part of the brain or central nervous system may get involved [5]. Studies have shown that PRES can occur in patients with a blood pressure considerably lower than the level required to produce hypertensive encephalopathy [6-7]. A retrospective review study [8] on 151 PRES patients has shown that the incidence of PRES was significantly higher in multiparous parturients with eclampsia at gestational ages greater than 36 weeks who had undergone an LSCS. Shaikh et al. also observed that the incidence was higher during the postpartum period [8].

Our patient presented to us at 38 weeks of gestation, and she had remained normotensive throughout her pregnancy. Initially, during the postoperative period, she presented with a parieto-occipital headache, which led to an initial diagnosis of post-dural puncture headache. Subsequently, she developed bilateral vision loss (which excluded CRAO as the probable cause) along with focal seizures.

PRES and RCVS share similar clinical features. However, the headache in RCVS is severe and thunderclap in nature and peaks within one minute, whereas PRES patients present with a gradual onset, mild to moderate, and self-limited type of headache [9].

In non-pregnant patients, moderate-to-severe hypertension is observed in approximately 70-80% of cases, out of which 20-30% of patients have normal or minimally elevated blood pressure [10]. However, to date, we do not have any study that can show a positive correlation between the severity of hypertension and the severity of the clinical or radiological features of PRES.

Burnet et al. observed in their study that approximately 70% of patients had seizures, whereas 3-13% of patients presented with status epilepticus [11]. In a significant number of studies, authors failed to report focal neurological signs in patients presented with PRES, and if all of them had been reported, the incidence would have been very low [4]. None of the studies have reported any specific symptoms that were seen exclusively in pregnant patients [4].

PRES is reversible only if it is managed within a short period of time; otherwise, it can lead to irreversible neurological damage or may even result in death [4]. Early detection and prompt management of PRES, therefore, are of utmost importance.

The mainstay of treatment for PRES in patients with preeclampsia is efficient management of pregnancy-induced hypertension [12]. Uncontrolled and persistently high blood pressure results in vasogenic as well as cytotoxic edema, which can lead to cerebral infarction and a permanent neurologic deficit. Antihypertensives, along with anti-edema agents and control of seizures, are therefore the cornerstones of the management of PRES. The termination of the pregnancy remains the definitive treatment [13].

Our patient presented with PRES during the postpartum period, and hence early intervention focused on monitoring vital parameters and obtaining MRI scans was crucial. Targeted reduction of cerebral edema was equally important, as it helped us to avoid neurological sequelae and also early and late complications. Whenever PRES is suspected, we must attempt to detect signs of the development of cytotoxic edema with a cerebral MRI scan [14-15]. The time interval between the seizure and the MRI scan influences the spread and localization of edema [2]. The use of apparent diffusion coefficient maps and diffusion-weighted MRI imaging can further increase the accuracy of the analysis of cerebral lesions in PRES [2].

## Conclusions

PRES should always be suspected when women have acute vision loss, seizures, and headaches during the peripartum period. In our case, we could diagnose PRES very early and successfully achieve a complete remission of symptoms. An MRI scan, being the gold standard for the diagnosis of PRES, should be done at the earliest. Prompt and efficient management of blood pressure, control of seizures, and prevention of cytotoxic edema are the cornerstones of the management of PRES and can avoid the development of irreversible neurologic injury.
